# Bridging Data, Semantics, and Clinical Reasoning: A Knowledge Graph Framework for Pediatric Obstructive Sleep Apnea

**DOI:** 10.3390/children13050602

**Published:** 2026-04-27

**Authors:** James D. Geyer, Jiaqi Gong, Paul G. Cox, Randi J. Henderson-Mitchell, Camilo R. Gomez, Adnan I. Qureshi, Shelby G. Branch, Sophia R. Geisser, Paul R. Carney

**Affiliations:** 1 Institute for Rural Health Research, University of Alabama, Tuscaloosa, AL 35487, USA; 2 University Medical Center Neurology and Sleep Medicine, University of Alabama, Tuscaloosa, AL 35487, USA; 3 Alabama Center for the Advancement of AI, Department of Computer Science, University of Alabama, Tuscaloosa, AL 35487, USA; 4 Evimero, LLC, Tuscaloosa, AL 35406, USA; 5 Department of Neurology, University of Missouri School of Medicine, Columbia, MO 65212, USA; 6 Department of Psychology, University of Alabama, Tuscaloosa, AL 35487, USA; 7 Sunstone Health, Boston, MA 02101, USA

**Keywords:** pediatric obstructive sleep apnea, artificial intelligence, knowledge graph

## Abstract

**Highlights:**

**What are the main findings?**

- Current artificial intelligence for pediatric sleep medicine is limited by fragmented data, a lack of clinical explainability, and failure to account for age-specific developmental differences.
- A proposed knowledge graph framework successfully integrates multimodal data including polysomnography, electronic health records, and clinical narratives, into a unified reasoning system.

**What are the implications of the main findings?**

- This framework enables the development of interpretable clinical decision tools that provide clinicians with auditable evidence for personalized screening and post-surgical risk assessments.
- Standardizing pediatric sleep data facilitates large-scale research collaborations and enables the projection of specialized care into underserved and rural regions.

**Abstract:**

Background/Objectives: Pediatric obstructive sleep apnea (OSA) is a complex disorder with a variable presentation and often challenging diagnostic testing. The history and physical examination in pediatric OSA frequently differ from those in adults. The treatment options are multifaceted and must be tailored to the individual patient. Artificial intelligence (AI) modalities currently employed in pediatric sleep medicine face several important limitations: modality fragmentation, lack of explainability, and limited semantic integration. Method: Our team proposes a new vision for AI and pediatric sleep medicine. This platform is based on a knowledge graph (KG) framework integrating structured and unstructured data to enable reasoning, personalization, and clinical decision support. Results: This framework represents a conceptual architecture; it has not yet been empirically implemented, and the use cases described herein are illustrative of its intended capabilities. Components of the infrastructure developed for similar applications have been successfully implemented. The quantitative feasibility pilot KG represented 100% multimodal data with >90% semantic completeness. Conclusions: Fully realized and deployed into the clinical space, this pediatric OSA KG system will enhance tertiary care programs and help project tertiary-level pediatric care into underserved regions.

## 1. Introduction

Obstructive sleep apnea (OSA) is defined by repeated episodes of partial or complete upper-airway obstruction during sleep. These episodes lead to disrupted sleep and abnormalities in gas exchange. The patient may or may not snore and is often unaware of the breathing changes [1]. The diagnosis of pediatric OSA is confirmed with overnight polysomnography (PSG), as recommended by the American Academy of Pediatrics (AAP), [2] American Academy of Sleep Medicine (AASM) [3], and European Respiratory Society (ERS) [4]; PSG measures the apnea-hypopnea index (AHI), defined as the number of apneas and hypopneas per hour of sleep. In children under 13 years of age, an AHI above 1 respiratory event/hour is considered abnormal. AHI classifies pediatric OSA severity into mild (1–4/h), moderate (5–9/h), and severe (≥10/h). These thresholds are lower than those for adults and reflect children’s greater sensitivity to airway obstruction. Adult OSA severity, which may be appropriate for assessment of adolescent OSA, is classified by AHI into mild (5–15/h), moderate (15–30/h), and severe (≥30/h) [5]. The AAP advises that primary care providers screen for snoring and other OSA symptoms at routine health visits. This screening is compromised by the complexity of history and physical examination findings in sleep disorders. A thorough and optimized OSA evaluation is needed for any child with snoring or other symptoms before considering therapeutic interventions like surgery or continuous positive airway pressure (CPAP).

**Epidemiology:** OSA is common in childhood. Studies report that approximately 2–5% of the pediatric population has OSA. The peak prevalence occurs in early childhood, typically in ages 2–8 years. This is often secondary to enlarged tonsils and adenoids [6,7]. In adolescents, OSA remains an important issue, but the underlying etiologies shift, such as obesity [2]. The prevalence of OSA in older adolescents (16–19 years) is approximately 4%, with obesity and male sex as significant risk factors in this age group [8]. In obese teens, OSA can be strikingly common, potentially 40 to 60% [9]. Unlike in younger children, adolescent OSA more closely resembles adult patterns in epidemiology, symptomatology, physical findings, and polysomnographic findings. During childhood, OSA affects boys and girls roughly equally in early years. However, adolescent males with additional risk factors are more likely to have OSA [10]. Overall, pediatric OSA is underdiagnosed. Increased clinician awareness and screening are needed to identify affected children early.

**Clinical Features and Symptoms:** The nighttime symptoms of pediatric OSA often alert families to the problem. Loud snoring is common but not required and is frequently accompanied by observed pauses in breathing or gasping, labored breathing, restless sleep with frequent position changes, night sweats, or even bedwetting (nocturnal enuresis). Some children with OSA adopt unusual sleeping positions (e.g., neck hyperextended) to keep the airway open. Mouth breathing and dry mouth are also common due to chronic airway obstruction. By morning, children with OSA may wake with headaches or a dry throat. During the daytime, the classic adult symptom of excessive daytime sleepiness is less common in young children; instead, many pediatric patients with OSA exhibit behavioral and cognitive symptoms. Overt sleepiness during the day is rare in younger children with OSA and tends to be seen more in adolescents or in obese children. Rather than appearing lethargic, school-aged children with untreated OSA may struggle with behavioral and neurocognitive issues that may closely resemble attention-deficit/hyperactivity disorder (ADHD) [11,12], increasing the risk of an incorrect diagnosis of ADHD and unnecessary stimulant therapy.

Against this backdrop, artificial intelligence (AI) has been explored to improve the detection, characterization, and management of pediatric OSA. In polysomnography (PSG) signal processing, convolutional and recurrent neural networks, and anomaly-detection methods have been developed to automate event detection, sleep staging, and arousal identification from multichannel physiological signals. These systems can reduce scoring time and inter-scorer variability, and they can unmask subtle temporal patterns (e.g., REM-predominant obstruction, positional dependence, or hypoventilation signatures) that inform risk and therapy selection. Beyond PSG, supervised machine-learning models using features extracted from the electronic health record (EHR) (e.g., demographics, anthropometrics, comorbid diagnoses, medications, and vital signs) have been trained to prioritize referrals, predict study positivity, or anticipate residual disease after surgery. Questionnaires such as the Pediatric Sleep Questionnaire (PSQ) [13] and fatigue/sleepiness scales have been mined with elastic-net, gradient-boosting, and calibration frameworks to refine screening thresholds and triage strategies. More recently, large language models (LLMs) have been utilized to summarize narrative sleep reports, harmonize impressions across providers, and answer clinician queries using textual context.

Despite these advances, current AI efforts are siloed by modality and task and therefore struggle to generalize across settings and populations. PSG-only models often ignore comorbid conditions and contextual factors (e.g., craniofacial morphology, oropharyngeal anatomy, allergic rhinitis, asthma) that shape risk and outcomes [14,15]. EHR-based risk scores can inherit coding biases, lack temporal granularity, and underperform in health systems with different documentation practices [16,17]. Questionnaire-centric models are sensitive to language, culture, and caregiver education [18]. Across modalities, explainability remains limited: many systems provide predictions without mechanistic pathways that clinicians can validate, eroding trust in high-stakes decisions [19]. Moreover, age-conditioned AHI thresholds, growth-related anatomical changes, and developmental stages are rarely embedded formally, which impairs model calibration across a child’s trajectory [20]. These gaps point to the need for an integrative, semantically grounded approach that unifies evidence, preserves provenance, promotes governance, and enables transparent reasoning. Use of LLMs alone exposes the system to unnecessary risks of hallucinations. LLMs, however, provide the structure for an excellent user interface.

A knowledge graph (KG) framework offers a coherent solution by representing pediatric OSA as a network of entities and relations grounded in clinical standards and enriched with temporal structure [21]. In such a KG, concepts such as apnea subtypes, age-specific severity thresholds, symptom clusters, comorbidities, interventions, and outcomes are encoded as typed nodes, linked by clinically meaningful relations (e.g., “has-risk-factor,” “treated-by,” “observed-in,” “responds-to”). Data from PSG, EHR, clinical notes, questionnaires, and wearables are mapped into this semantic scaffold with explicit timestamps, confidence scores, and provenance [22]. Because the KG aligns to ontologies such as SNOMED CT, LOINC, RxNorm, and HL7 FHIR resource models, it can interoperate with clinical systems, support federated analytics across institutions, and allow fine-grained validation via shape constraints [23]. Crucially, the graph becomes not just a repository but a reasoning substrate: pathway queries can retrieve evidence chains from symptom to intervention to outcome, graph algorithms can identify similar patient trajectories, and temporal rules can formalize definitions like “residual OSA six months after adenotonsillectomy” [24,25].

The conceptual architecture we advance has four pillars. First, a multi-granular semantic layer captures anatomical, physiological, behavioral, and demographic features relevant to pediatric OSA, enabling phenotype definitions that respect age and developmental stage. Second, a multimodal data integration pipeline ingests structured PSG features (AHI components, arousals, oxygen nadirs, REM-specific indices), EHR elements (ICD/SNOMED problems, medications with RxNorm codes, vitals and anthropometrics with LOINC/UCUM units), unstructured narratives from sleep reports and clinic notes, standardized questionnaire responses, and home-context wearable signals [2]. Third, a temporal model binds discrete encounters into episodes: screening, diagnostic testing, intervention, and follow-up, with start/stop times and outcome windows supporting longitudinal analyses of recurrence, growth-related changes, and treatment durability. Fourth, an interoperability layer aligns graph entities and value sets with SNOMED and FHIR, enforces data-quality constraints, and exposes standards-based APIs for clinical decision support and research [26].

Building on this architecture, we critically review the current landscape and its gaps. PSG algorithms excel at event-level accuracy but rarely encode why patterns matter clinically, for example, by connecting REM-predominant obstruction to neurocognitive outcomes or surgical decision-making [27]. EHR risk models are often trained on limited, single-center cohorts and lack explicit semantics for pediatric nuances, leading to drift when deployed elsewhere [28]. Questionnaire-based tools can be valuable triage instruments but need integration with objective measures and demographic context to avoid misclassification [29]. LLM-based summarization and question answering show promise but require grounding to verifiable facts to prevent hallucinations. A KG-centric approach addresses each shortcoming by (a) integrating modalities under shared semantics, (b) preserving audit trails from raw data to derived assertions, (c) enabling explainable paths that clinicians can inspect, and (d) supporting pediatric-specific rules that adjust as a child matures [24].

We further propose using cases and a roadmap for future research that operationalizes the KG vision [30]. In clinical decision support, the graph can power personalized screening by combining questionnaire scores with EHR comorbidities and growth metrics, flagging high-risk children even when PSG capacity is constrained [31]. For peri-operative care, temporal graph rules can estimate the risk of residual OSA after adenotonsillectomy as a function of baseline AHI, obesity status, and craniofacial findings, guiding follow-up testing [32]. In longitudinal management, the KG can monitor trajectories, linking medication adherence, allergy control, and weight change to symptom resolution or recurrence, and prompt timely interventions [33]. For research, the KG enables reproducible cohort identification (e.g., “children under 10 with REM-predominant severe OSA and asthma who received CPAP”), supports hypothesis generation around comorbidity clusters and environmental exposures, and facilitates multi-site studies through ontology-aligned data sharing. Finally, when coupled with LLMs through retrieval-augmented generation constrained by graph facts and shape validations, the system can produce trustworthy patient summaries and evidence-linked answers to clinician questions. At the same time, feedback loops convert clinician edits into ontology refinements and improvements to extraction.

In summary, pediatric OSA is prevalent, impactful, and systematically under-detected due to heterogeneous presentations and fragmented data. AI has advanced individual components such as signal processing, risk modeling, and narrative understanding, but lacks the semantic coherence, generalizability, and explainability required for dependable clinical adoption. A knowledge graph framework unifies modalities within a pediatric-aware semantic layer, encodes temporal disease courses, and anchors LLM-enabled services to verifiable facts. The contributions of this paper are threefold: it articulates a conceptual architecture for a pediatric OSA KG; it offers a critical appraisal of current AI components and the gaps that hinder their clinical utility; and it outlines concrete use cases and a research roadmap to translate this framework into real-world decision support and multicenter research infrastructure. Through collaborative development with clinicians, informaticians, and standards bodies, this approach can help deliver earlier diagnosis, more precise treatment selection, and equitable access to high-quality pediatric sleep care.

With iterative refinement utilizing large datasets, the KG approach is expected to outperform current state-of-the-art multimodal AI models in clinical utility, explainability, and reliability because it is based on a structured, symbolic, and evidence-based system. Current AI models excel at detecting patterns across both text and images, but they frequently struggle with medical reasoning, hallucinations, explainability, and transparency. KGs address these problems by anchoring the platform in verified medical knowledge.

## 2. Background and Related Work

### 2.1. Pediatric Obstructive Sleep Apnea

Pediatric obstructive sleep apnea (OSA) arises from a confluence of anatomic, physiologic, developmental, and environmental factors that narrow or destabilize the upper airway during sleep. Etiologically, adenotonsillar hypertrophy is the dominant driver in early childhood, often interacting with craniofacial phenotypes such as midfacial hypoplasia, retrognathia, high-arched palate, and dental crowding, which reduce retropalatal or retroglossal airway caliber [34,35]. In later childhood and adolescence, obesity contributes via fat deposition around the pharyngeal airway and altered ventilatory control; neuromuscular conditions that diminish pharyngeal dilator tone further increase collapsibility [36,37,38]. Allergic rhinitis and chronic nasal obstruction increase inspiratory resistance and promote mouth breathing, which can worsen airway patency and disrupt sleep. These mechanisms are modulated by maturation of sleep architecture, circadian influences on ventilatory control, and growth-related changes in craniofacial structures, yielding a heterogeneous set of phenotypes that evolve across developmental stages [39,40].

Symptoms reflect this heterogeneity and often diverge from those of adults. At night, caregivers may report loud snoring, witnessed apneas or gasps, labored or paradoxical breathing, restless sleep with frequent position shifts, diaphoresis, and enuresis [41]. The presentation is highly variable, with all, a few, or even none of these symptoms. Children sometimes adopt compensatory postures (e.g., neck hyperextension) to keep the airway open [42]. Morning complaints include a sore throat and a headache. Daytime manifestations in younger children tend to involve behavioral and cognitive sequelae, such as hyperactivity, irritability, inattention, executive dysfunction, and poor school performance, rather than frank sleepiness [43,44]. By contrast, adolescents (particularly with obesity) more often exhibit classic excessive daytime sleepiness. Because these manifestations are nonspecific and vary with age, pediatric OSA is frequently underrecognized without systematic screening [2].

Comorbid conditions are common and clinically important. Asthma and allergic rhinitis can exacerbate upper-airway inflammation and resistance, creating bidirectional symptom amplification [45,46]. Hypertension and early vascular dysfunction have been observed in children with moderate-to-severe OSA, likely mediated by intermittent hypoxia, sympathetic activation, and endothelial injury [47]. Obesity not only elevates OSA risk but also attenuates responses to first-line therapies and increases the likelihood of residual disease after surgery [48]. Neurobehavioral conditions, including attention-deficit/hyperactivity disorder (ADHD), may be mimicked or worsened by sleep-disordered breathing; conversely, treating OSA can improve attention and behavior in some children [49]. Recurrent tonsillitis often coexists with adenotonsillar hypertrophy and may influence the timing and rationale for adenotonsillectomy [50,51]. Recognizing and actively managing these comorbidities is integral to effective OSA care.

Diagnostic pathways center on objective sleep testing supported by clinical examination and standardized questionnaires. In-laboratory polysomnography (PSG) remains the reference standard in pediatrics, providing event-level annotation (apneas, hypopneas, arousals), oxygen saturation dynamics, carbon dioxide measures, sleep staging, body position, and limb movements [52]. Pediatric scoring rules differ from adult criteria and must be applied with attention to age-specific definitions and AHI thresholds. By contrast, home sleep apnea tests are generally not indicated for children because of reduced sensitivity for hypoventilation, challenges with sensor adherence and artifact in smaller patients, and the need for comprehensive physiologic monitoring to guide therapy [53]. The physical examination often reveals signs of chronic upper-airway obstruction and, in some cases, neuromuscular contributors to airway weakness. Nasal evaluations may show turbinate hypertrophy, septal deviation, or allergic stigmata; the oropharyngeal exam may reveal enlarged tonsils, elongated soft palate, or a high-arched palate; neck assessment may identify truncal/neck adiposity or masses; craniofacial assessment considers mandibular/maxillary relationships, midface hypoplasia, dental crowding, and bite class. Mouth breathing, adenoidal facies, and growth parameters provide additional context. Standardized questionnaires complement objective testing; the Epworth Sleepiness Scale for Children and Adolescents (ESS-CHAD) [54] and instruments such as the Pediatric Sleep Questionnaire (PSQ) [13] quantify symptom burden and can prioritize referrals, though they do not replace PSG.

Treatment is individualized and longitudinal. Adenotonsillectomy is first-line therapy for most children with adenotonsillar hypertrophy and clinically significant OSA, but residual disease is common in those with obesity, craniofacial anomalies, or severe baseline OSA. Continuous positive airway pressure (CPAP) is indicated when surgery is contraindicated, declined, or ineffective; its success hinges on careful mask fitting, desensitization, behavioral support, and ongoing adherence monitoring [55]. Oral appliances are not typically used in pediatric OSA because of ongoing craniofacial growth, though select orthodontic interventions (e.g., rapid maxillary expansion in appropriately selected phenotypes) may have a role within multidisciplinary care [56]. Weight management, allergy control with intranasal steroids or leukotriene modifiers, and positional therapy can be useful in specific contexts [57]. Regardless of initial therapy, structured follow-up is essential to reassess symptoms, monitor growth and weight trajectories, and repeat PSG when residual disease is suspected or clinical status changes [58].

### 2.2. Existing AI Approaches

Artificial intelligence has been deployed across the pediatric OSA pathway to improve detection, characterization, and care coordination, with the densest activity in PSG signal processing. Convolutional neural networks (CNNs) and temporal models (e.g., bidirectional LSTMs, temporal convolutional networks, transformers) have been trained on multi-channel PSG to automate sleep staging and event detection, reduce inter-scorer variability, and surface patterns that are challenging to quantify manually (REM-predominant obstruction, positional dependence, hypoventilation signatures) [59,60]. Self-supervised and anomaly-detection approaches leverage large volumes of unlabeled signals to pretrain robust feature extractors that adapt to pediatric physiology and smaller labeled datasets [61]. Beyond signals, clinical risk models ingest EHR-derived features and questionnaire scores to prioritize referrals, predict PSG positivity, estimate residual OSA risk after adenotonsillectomy, or recommend perioperative monitoring [20,62,63]. Feature selection techniques (e.g., LASSO, mutual information, Boruta) and ensemble learners (gradient boosting, random forests, stacking) are commonly used, with calibration methods to improve clinical interpretability [64]. More recently, large language models (LLMs) have been piloted to summarize narrative sleep reports and clinic notes, harmonize impressions across providers, extract structured entities and relations from unstructured text, and support question answering for clinicians and families.

Despite promising results, key limitations persist. Most systems are confined to a single modality (signals, tabular EHR, or text) and a narrow task, which restricts generalizability across institutions and patient subgroups [65]. Pediatric-specific semantics, such as age-conditioned AHI thresholds, growth-related anatomy, and developmentally appropriate symptom constructs, are rarely encoded explicitly, thereby impairing calibration across developmental stages. Label scarcity and domain shift (different sensors, scoring rules, and documentation practices) challenge model robustness; class imbalance (rarer severe phenotypes) and covariate shift (obesity prevalence, referral patterns) further degrade transportability [66]. Explainability is often limited to post hoc feature attributions that do not articulate causal or mechanistic pathways clinicians can validate [19]. Finally, provenance tracking is inconsistent, complicating audit and regulatory evaluation [67]. These gaps motivate integrative approaches that unify modalities, embed pediatric semantics, and provide transparent, provenance-aware reasoning.

### 2.3. Knowledge Graphs in Clinical AI

Knowledge graphs (KGs) offer a principled foundation for such integration by representing clinical entities and their relationships in a computable, standards-aligned form. Examples of widely used clinical resources include SNOMED CT for problems and findings, the Unified Medical Language System (UMLS) for cross-terminology mappings, and the Observational Health Data Sciences and Informatics (OHDSI) common data model, which demonstrate how semantic harmonization enables multi-institutional analyses and reproducible phenotyping [68]. Research-oriented graphs such as MIMIC-KG illustrate how time-stamped observations, interventions, and outcomes can be linked to support cohort discovery, outcome modeling, and hypothesis generation [69]. The strengths of KG-based approaches include semantic integration across heterogeneous sources; explicit provenance and versioning that trace each assertion to its data source, timestamp, and transformation; and powerful query ability via graph languages (SPARQL/Cypher) and graph algorithms (similarity search, community detection, path reasoning).

When coupled to machine learning, graphs can inform feature construction (graph embeddings), support label-efficient learning (distant supervision), and constrain generation in LLM-based systems through retrieval-augmented pipelines that ground outputs in verifiable facts.

However, important gaps remain for pediatric OSA. Pediatric-specific ontologies are sparse, and general terminologies often lack the age-conditioned definitions and developmental nuances central to pediatric sleep medicine [70]. Multimodal integration is limited: few graphs natively integrate high-frequency physiologic signals (PSG), tabular EHR data, questionnaires, and narrative text with consistent temporal alignment and high-quality metadata [71]. Temporal reasoning itself is underdeveloped. Most graphs represent time as static attributes rather than as first-class structures that capture episodes, intervals, and event orderings necessary for trajectory-aware inference (e.g., defining residual OSA within a specific post-operative window) [72]. Addressing these gaps requires a pediatric-first KG that encodes age-specific severity thresholds and symptom constructs; ingests signals, tabular data, and narratives with synchronized timelines and uncertainty measures; and enforces provenance and validation constraints. Such a resource can serve as the semantic backbone for explainable, portable, and clinically trustworthy AI systems across the pediatric OSA continuum [73].

## 3. Materials and Methods

### 3.1. A Knowledge Graph Framework for Pediatric OSA

**Figure 1:**
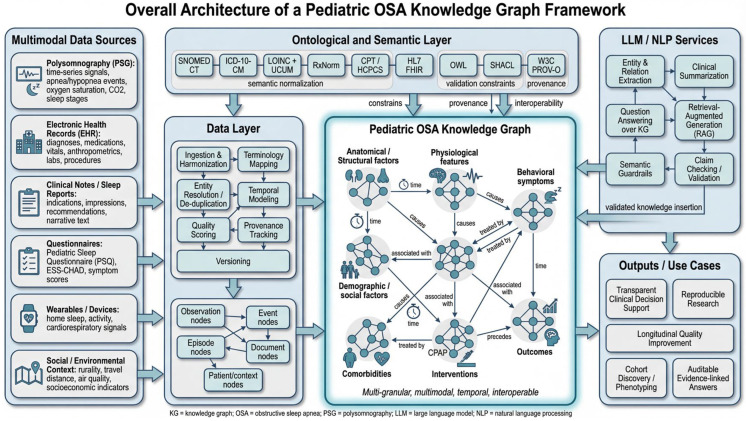
Layered architecture of the pediatric OSA knowledge graph framework, showing how multimodal data are harmonized through ontology-aligned pipelines and connected to LLM/NLP services under semantic constraints.

This section lays out a practical, implementation-ready framework for a pediatric OSA knowledge graph (KG). The objective is to fuse heterogeneous clinical and contextual data into a semantically coherent substrate that supports transparent decision support, reproducible research, and longitudinal quality improvement [21]. The design emphasizes four pillars: multi-granularity, multimodality, temporality, and interoperability, which are implemented through a layered architecture comprising an ontological layer, a data layer, and LLM/NLP services bound to the KG by semantic constraints [74]. Figure 1 provides an overview of this architecture, illustrating how multimodal pediatric OSA data are harmonized into a standards-aligned knowledge graph and linked to constrained LLM/NLP services for explainable downstream use.

### 3.2. Core Design Principles

**Multi-granular.** Pediatric OSA emerges from interactions across anatomical structure, physiology, behavior, and demographics [75]. The KG therefore represents entities and attributes at multiple levels of abstraction [75]. At the anatomical/structural tier, classes capture craniofacial morphology (e.g., mandibular retrognathia, midface hypoplasia), adenotonsillar size, nasal patency, and airway caliber [76]. This anatomic layer can be supported by vision AI with automated analysis of the airway and facial structures. The physiological tier stores PSG-derived measures such as AHI and its components (apnea index, hypopnea index), arousal index, oxygen nadir, end-tidal/transcutaneous CO_2_, sleep stage architecture, respiratory effort surrogates, and periodicity of events. The behavioral tier includes snoring frequency, witnessed apneas, parasomnias, bedwetting, and teacher/parent-reported attention or hyperactivity concerns [77]. The demographic tier encodes age (gestational and chronological), sex, growth parameters, race/ethnicity (when appropriate and consented), and social determinants (insurance type, language, distance to specialty care, and other barriers to care). Multi-granularity allows the graph to support both fine-grained mechanistic questions (e.g., how end-tidal CO_2_ trajectories relate to arousals) and higher-level clinical reasoning (e.g., whether adenotonsillectomy resolves behavioral symptoms in specific phenotypes) [38,78].

**Multimodal.** The framework integrates polysomnography (PSG), electronic health records (EHR), clinical notes, validated questionnaires, and wearable/device data. PSG contributes time-series events and aggregated features; EHR contributes diagnoses, medications, vitals, anthropometrics, laboratory results, and procedures; narrative notes add nuanced clinical impressions and rationales; questionnaires such as the Pediatric Sleep Questionnaire (PSQ) and ESS-CHAD encode standardized symptom burdens; and wearables provide home-context sleep/wake and cardiorespiratory signals [52,53]. Modality-specific uncertainty is preserved as first-class metadata to avoid over-confidence when modalities disagree [79].

**Figure 2:**
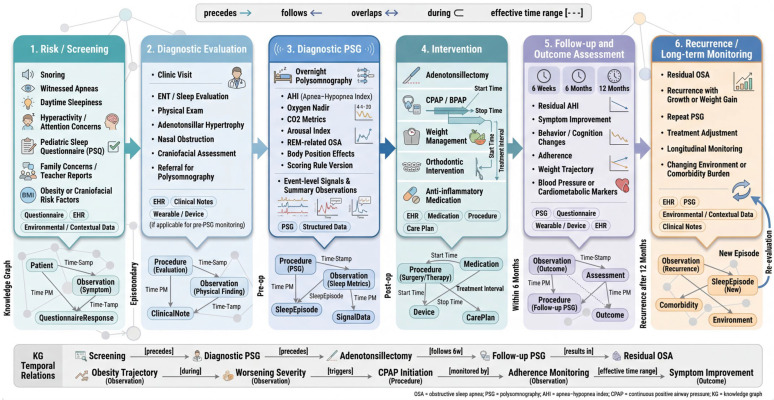
Temporal modeling of pediatric OSA episodes across screening, diagnosis, treatment, follow-up, and recurrence, enabling longitudinal reasoning over interventions and outcomes.

**Temporal.** Pediatric OSA trajectories evolve with growth, weight change, surgery, orthodontic development, and comorbidity management [79]. The KG models time explicitly: clinical “episodes” (screening → diagnostic PSG → intervention → follow-up → recurrence) are linked by temporal relations (precedes/follows, overlaps, during). Observations carry effective time ranges; interventions carry start/stop times and dose/exposure details; outcomes are measured at defined windows (e.g., 6-week, 6-month, 12-month follow-ups) [52]. Figure 2 illustrates this temporal patient journey and shows how multimodal observations, interventions, and outcomes are anchored to longitudinal episodes rather than treated as isolated clinical snapshots. This enables longitudinal queries (e.g., residual OSA six months after adenotonsillectomy in children with obesity) and supports causal modeling and survival analysis layered atop the graph.

**Interoperable.** To ensure portability and safety, entities are mapped to clinical standards. Conditions, findings, and procedures align to SNOMED CT; diagnoses to ICD-10-CM (with pediatric-appropriate use); observations to LOINC with UCUM units; medications to RxNorm; and care processes to CPT/HCPCS where relevant [80]. Data exchange is mediated via HL7 FHIR resources (e.g., Patient, Encounter, Observation, Condition, Procedure, Medication Statement, Device, QuestionnaireResponse). Interoperability allows federated learning across institutions and simplifies regulatory review, audit, and integration with clinical systems [81].

### 3.3. Ontological Layer

The ontology provides the semantic backbone. **Concepts** include apnea types (obstructive, central, mixed, hypopnea per scoring rule), severity categories (age-appropriate AHI thresholds), symptom clusters (nocturnal vs. daytime behavioral/cognitive), comorbidities (obesity, asthma, allergic rhinitis, craniofacial anomaly, neuromuscular disease, hypertension, ADHD), and interventions (adenotonsillectomy, CPAP/BPAP, weight management, orthodontic therapies, anti-inflammatory regimens) [14]. Severity classes are defined with pediatric-specific cut points and may differ for adolescents approaching adult criteria; these distinctions are encoded as age-conditioned class axioms rather than static labels [82].

**Relationships** capture clinically meaningful semantics, including *has-risk-factor* (e.g., obesity → OSA) [52], *treated-by* (severe OSA → CPAP) [83], *observed-in* (hypoventilation → REM sleep), *responds-to* (behavioral symptoms → adenotonsillectomy), *contraindicated-with* (specific craniofacial anomalies → oral appliances), *co-occurs-with* (OSA ↔ asthma), *worsened-by* (upper respiratory infection → OSA severity) [84], and *precedes/follows* (pre-op PSG → surgery → post-op PSG). Temporalized relations allow reasoning like “post-operative AHI < 2 within 6 months [85]”.

The Shapes Constraint Language (SHACL) is a language for validating Resource Description Framework (RDF) graphs against a set of conditions. SHACL has a core specification, extensions for related query languages, User Interface generation, rule-based reasoning and a data profiling mechanism. SHACL models are defined in terms of constraints on the content, structure, and meaning of a graph. Wherever possible, the ontology **leverages existing pediatric vocabularies and meta-thesauri** (e.g., SNOMED CT hierarchies for pediatric findings, UMLS mappings, FHIR ValueSets for pediatric vitals) and extends them minimally to cover OSA-specific constructs not well represented (e.g., child-specific scoring variants, questionnaire score semantics) [52,86]. Constraints are formalized using OWL restrictions for taxonomic reasoning and SHACL shapes for data validation (e.g., a *PSGObservation* must reference a LOINC code and UCUM unit, and an *AHI* value must be associated with a scoring rule version) [79]. Provenance is modeled with W3C PROV-O so that every edge and attribute can be traced to its data source, timestamp, and transformation step, enabling auditability and trust [13].

The ontology layer manages the transition from infant to pediatric to adult sleep physiology and associated diagnostic assessments via a structured approach that maps dynamic developmental changes such as the definition of AHI which varies by age into a structured framework. To ensure clinical utility and explainability, this involves moving from static, stage-specific ontologies to a “developmental continuum” model that uses shared semantic anchors (e.g., SNOMED-CT, LOINC) to bridge pediatric and adult data systems.

### 3.4. Data Layer

The data layer operationalizes ontology-aligned ingestion, harmonization, and storage.

**Polysomnography (structured features).** The pipeline extracts per-study aggregates (AHI and sub-indices, arousal index, oxygen nadir, REM-specific indices, CO_2_ metrics), per-event sequences (start/stop, type, stage, body position), and channel-level quality indicators. Signal-processing metadata (scoring rule version, scorer identity, automated vs. manual adjudication) is preserved. Features are stored both as time-stamped events linked to *SleepEpisode* and as summary *Observation* nodes with LOINC/UCUM annotations, enabling analyses from both event-level and study-level perspectives [78].

**EHR (ICD, medication, vitals, demographics).** Diagnoses (e.g., OSA, obesity, asthma), procedures (adenotonsillectomy, orthodontic interventions), medications (intranasal steroids, leukotriene antagonists), anthropometrics (height, weight, BMI z-scores), and vitals (blood pressure percentiles) are normalized to SNOMED/ICD/RxNorm/LOINC and linked to encounters and episodes. Growth trajectories are modeled as sequences to support phenotype definitions that depend on temporal change (e.g., a rapid increase in BMI percentile preceding OSA onset).

**Sleep study reports (unstructured).** Narrative sections (“Indications,” “Impression,” “Recommendations”) often contain nuanced rationales and clinical caveats. Reports are stored as documents with stable identifiers; LLM/NLP services extract entities (e.g., “REM-predominant OSA,” “positional dependence”) and relations (e.g., “surgery recommended because…”) into the KG with confidence scores and provenance backpointers to text spans, enabling human verification [52,53].

**Survey/questionnaire data (e.g., PSQ).** Raw item responses and total/subscale scores are modeled explicitly, with score interpretations (e.g., cutoffs indicating high risk) encoded as computable rules. This allows queries such as “children with high PSQ score but normal in-lab AHI” to investigate discordant cases [79].

**Social/environmental data (e.g., rurality, pollution).** Geocoded features (appropriately de-identified and privacy-protected) capture rural–urban classification, travel distance/time to sleep centers, local air quality indices, secondhand smoke exposure proxies, and socioeconomic indicators. These are linked to patients via time-varying residence nodes so environmental changes (e.g., seasonality in pollution) can be studied in relation to symptoms and PSG metrics [87].

**Architecture and storage.** A hybrid approach supports both semantic reasoning and high-performance analytics: an RDF/OWL triple store with SPARQL endpoints provides standards-based semantics and reasoning, while a property-graph mirror (e.g., Neo4j) accelerates path queries and graph algorithms (similar patient retrieval, community detection). ETL pipelines enforce SHACL constraints, perform de-duplication and entity resolution, and attach data quality scores (completeness, plausibility, consistency). Versioning enables “time-travel” analyses and reproducibility (e.g., rerunning a cohort definition against the KG as of a specific date) [52,79].

### 3.5. LLM and NLP Integration

LLMs and classical NLP are coupled with the KG to extract knowledge, summarize context, and support question answering, always under **graph-aware constraints** to preserve clinical fidelity.

**Entity and relation extraction.** A hybrid pipeline combines dictionary/ontology lookups, weakly supervised sequence labeling, and LLM few-shot prompting to extract entities (diagnoses, symptoms, measurements, recommendations) and relations (“treated-by,” “observed-in,” “worsened-by”) from sleep reports, clinic notes, and literature. Extracted candidates are normalized to controlled vocabularies, disambiguated using KG context (e.g., distinguishing “central apnea” as an event vs. a diagnosis), and inserted only when SHACL constraints pass. Each assertion carries confidence, provenance (document, section, sentence offsets), and model version, enabling selective human review for low-confidence triples [88].

**Clinical summarization.** For point-of-care use, LLMs generate concise, structured summaries of a patient’s OSA trajectory (indications, PSG findings, comorbidities, prior interventions, outcomes). Summaries are composed by retrieving relevant subgraphs (patient → episodes → observations/interventions) and rendering them via controlled templates augmented by LLM natural-language fluency [83]. Consistent with the temporal patient journey shown in Figure 2, these summaries are organized around linked longitudinal episodes rather than isolated encounters, allowing clinicians to interpret findings in the context of disease progression, treatment response, and recurrence. KG facts are canonical and cannot be altered by the generator; where uncertainty exists (e.g., conflicting scores), the summary explicitly flags it and links to source nodes for drill-down inspection by the clinician [53].

**Question answering over the KG.** User queries are translated into graph operations through a retrieval-augmented generation (RAG) layer. First, the system retrieves a minimal subgraph satisfying the information need (e.g., “children < 10 years with severe OSA who had adenotonsillectomy and residual AHI ≥ 5 within 12 months”); second, SPARQL/Cypher executes the query; third, the LLM verbalizes results with explanation and citations to graph nodes/edges. “Semantic guardrails” constrain the LLM only to reference retrieved graph facts; responses include evidence links and uncertainty qualifiers [79]. For multi-site deployments, the RAG layer supports privacy-preserving federation by shipping queries to local KGs and aggregating de-identified results.

**Figure 3:**
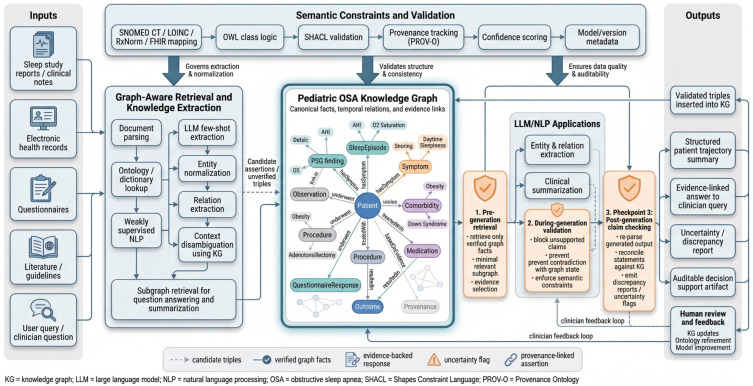
Knowledge-graph-constrained LLM/NLP workflow for extraction, summarization, and question answering with semantic guardrails and auditability.

**KG-constrained accuracy.** To minimize hallucinations and support regulatory-grade reliability, LLM/NLP components are tightly governed by the pediatric OSA knowledge graph and its semantic control layer throughout extraction, summarization, and question answering. As illustrated in Figure 3, the workflow begins with graph-aware retrieval and knowledge extraction, in which candidate entities and relations from notes, reports, questionnaires, and other inputs are normalized to controlled vocabularies, disambiguated using KG context, and treated as candidate assertions rather than trusted facts. Generation is bound to KG constraints at three checkpoints: (1) pre-generation retrieval, which limits the model’s context to verified graph facts and a minimal evidence-bearing subgraph [89]; (2) **during-generation validation**, which blocks unsupported claims and prevents outputs that would contradict graph state, semantic mappings, or SHACL constraints; and (3) **post-generation claim checking**, which re-parses the generated text, reconciles each statement against the KG, and emits discrepancy reports or uncertainty flags when inconsistencies are detected [90]. Only validated assertions are inserted back into the KG with provenance, confidence scores, and model-version metadata, ensuring that the graph remains the canonical source of truth. Continuous learning loops capture clinician feedback on extracted triples, summaries, and answers, converting corrections into training data and ontology updates.

**Equity Auditing and Bias Mitigation.** To ensure that the proposed KG framework supports equitable clinical decision-making, we incorporated a dedicated equity auditing and bias mitigation layer into the platform. Demographic and social determinant variables (e.g., race/ethnicity, insurance status, language, and geographic access to care) are explicitly represented as nodes and attributes within the KG, enabling subgroup-level analyses. Model outputs derived from the KG are evaluated using fairness-aware metrics, including subgroup-specific sensitivity, specificity, calibration, and disparity measures. To enforce structural and semantic integrity, we apply SHACL validation rules that detect imbalances in data representation, flag disproportionate associations between clinical recommendations and demographic attributes, and ensure that inferred relationships meet predefined equity constraints. In addition, the KG’s built-in provenance model enables traceability of each inference to its underlying data sources, supporting transparent bias auditing. When disparities are identified, mitigation strategies, including reweighting, stratified resampling, and ontology refinement, are applied iteratively, with clinician (human-in-the-loop) oversight, to reduce bias and improve model fairness. This approach ensures that the KG framework not only integrates multimodal data but also actively monitors and allows for mitigation of systemic inequities present in historical EHR data and other databases.

**Validation Roadmap.** The validation strategy for AI analysis of obstructive sleep apnea (OSA) involves several key steps to ensure the accuracy and reliability of the AI models. Accurate and reliable Training Datasets are of paramount importance. The large internal data repositories available to this group will be leveraged as well as open-access datasets such as the ISRUC-Sleep from the Sleep Medicine Centre of the Hospital of Coimbra University. Clinical validation, conducted in the clinical setting, is planned for future trials in the University Medical Center Sleep Medicine Clinics. The quality and completeness of the training and validation datasets available from the internal datasets has already been assessed.

**Model performance.** Transparency, explainability, provenance, and governance metrics for the datasets are available. These datasets are fully anonymized to preserve patient privacy with supervision by the Institutional Review Board.

**Figure 4:**
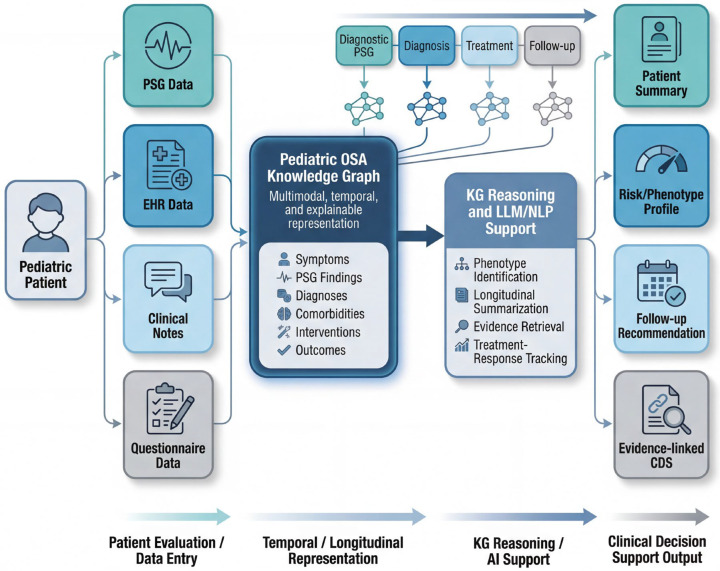
High-level flow of a single pediatric OSA patient journey from multimodal data capture to knowledge graph-enabled clinical decision support.

**Summary.** The proposed KG framework transforms pediatric OSA data into a computable, explainable, and standards-aligned representation that reflects the condition’s developmental and multimodal complexity [21]. The patient journey is summarized in Figure 4. Multi-granularity supports rich phenotype definitions; multimodality preserves complementary evidence; temporal modeling enables trajectory-aware reasoning; and interoperability ensures integration with clinical systems and research networks [72,81,91]. An ontology grounded in pediatric practice and reinforced by OWL/SHACL provides semantic rigor, while the data layer delivers robust pipelines for heterogeneous sources, quality control, and analytics [92]. Finally, LLM/NLP components tethered to the KG through RAG and semantic guardrails unlock scalable extraction, trustworthy summarization, and auditable question answering, positioning the framework for real-world clinical decision support and multicenter research [90,93].

## 4. Results

### 4.1. Use Cases and Applications

The pediatric OSA knowledge graph (KG) enables a family of applications that translate heterogeneous data into timely, explainable decisions and reproducible science [94]. By encoding symptoms, measurements, diagnoses, interventions, and outcomes as typed nodes with clinically meaningful relations and timestamps, the KG becomes both a unifying data substrate and a reasoning layer. As this is a proposed conceptual framework without empirical implementation to date, the following use cases are illustrative of the framework’s intended capabilities and are grounded in analogous work in the broader clinical informatics literature. Below, we detail how this foundation powers clinical decision support, accelerates research and discovery, and enables interpretable AI models designed for pediatric care [20,66].

### 4.2. Clinical Decision Support

In personalized screening, the KG fuses questionnaire responses with EHR context to estimate a child’s likelihood of clinically significant OSA before polysomnography. Questionnaire items (for example, PSQ subscales or ESS-CHAD scores) are represented as first-class observations linked to encounters, while the EHR contributes demographics, anthropometrics (BMI-for-age z-scores), comorbidities (asthma, allergic rhinitis, ADHD), medications (intranasal steroids, leukotriene modifiers), prior procedures, and social determinants (distance to a sleep center, insurance type) [15,57,95]. Graph queries assemble a patient-specific subgraph; a screening model then operates on graph features rather than flat tables, preserving age-conditioned semantics (e.g., pediatric AHI thresholds) and temporality (e.g., recent weight gain). The system returns a calibrated risk estimate, the top evidence paths supporting that estimate (such as “high PSQ snoring score + obesity + allergic rhinitis”) [15], and next-step options tailored to local capacity—watchful waiting with allergy control, expedited PSG referral, or pre-test counseling for families [96]. Decision curve analysis and impact metrics (positive predictive value at fixed referral slots, missed-case rate) guide threshold selection, enabling clinics to match screening to resource constraints while maintaining equity [97,98].

For postoperative risk assessment, the KG tracks the full adenotonsillectomy episode—preoperative PSG features (baseline AHI, oxygen nadir, REM-predominant or positional dependence), perioperative factors, and longitudinal follow-up findings [99]. Temporal relations bind these to outcome windows (e.g., residual OSA within 6–12 months) [32]. A predictive model on the graph estimates residual OSA risk conditional on phenotype (obesity, craniofacial anomaly, asthma control) and recommends surveillance strategies, such as timing for repeat PSG or prioritizing CPAP titration [33,100]. The output is accompanied by explanation subgraphs that show how features combine “severe baseline OSA + obesity + persistent allergic rhinitis → high residual risk” and by actionable levers (optimize allergy therapy; intensify weight management; plan earlier post-op testing). Because provenance is explicit, clinicians can audit which measurements, rules, and sources contributed to each recommendation.

A sleep-specialist assistant layers case-based reasoning atop the KG. When presented with a new patient, the assistant retrieves clinically similar trajectories using graph similarity (embedding-based nearest neighbors constrained by age and key comorbidities) and surfaces outcomes of comparable cases, linked guidelines, and evidence [101]. For example, for an 8-year-old with REM-predominant OSA, obesity, and asthma, the assistant may display cohorts where adenotonsillectomy improved AHI but residual disease persisted in obese children [78,99], along with comparative outcomes for early CPAP initiation versus staged management [100]. The assistant’s narrative is generated through retrieval-augmented generation constrained by graph facts, ensuring that all statements are grounded in verifiable nodes and edges and are accompanied by citations and confidence levels [102].

### 4.3. Research and Discovery

The KG streamlines hypothesis generation by enabling population-level patterns to be explored without bespoke data wrangling. Investigators can query age-stratified AHI distributions, examine REM-predominant patterns by sex or obesity status [10], or test associations between comorbidity clusters (asthma + allergic rhinitis) and oxygen desaturation burden [103,104]. Because temporal intervals are explicit, analyses can differentiate pre- and post-intervention trajectories and evaluate seasonality (e.g., allergy peaks) or growth-related changes [105]. Equity-focused questions such as differences in access to PSG or time-to-treatment by rurality or insurance are likewise computable, with sensitivity analyses facilitated by provenance and data-quality scores [95,98].

Cohort identification becomes reproducible and portable. Phenotypes are formalized as graph patterns—for instance, “children < 10 years, baseline AHI ≥ 10, REM-predominant events, comorbid asthma, underwent adenotonsillectomy, residual AHI ≥ 5 within 12 months.” These patterns can be versioned, shared, and re-executed across institutions that map their data to the same ontology, enabling multi-site studies without reinventing inclusion logic [106,107]. Graph embeddings complement rule-based phenotypes by clustering children with similar disease trajectories, helping researchers discover latent subtypes [108,109] (e.g., positional-dominant OSA with normal BMI versus obesity-driven OSA with hypoventilation features) that might merit different follow-up [110].

Augmented literature review and knowledge synthesis leverage the KG’s ontology to index publications and guideline statements by the concepts they address (apnea subtype, intervention, age band, comorbidity) [111]. An LLM retrieves graph-linked evidence to answer targeted questions: “What is the expected change in AHI after adenotonsillectomy in obese children under 10?” and produces an evidence table with citations, effect sizes, and study contexts [102,112]. Contradictions across studies are flagged when graph-encoded populations or outcome definitions differ, and new papers are incrementally integrated by extracting entities and relations with provenance back to text spans [113]. The result is a living, pediatric-aware evidence graph that reduces duplication and accelerates consensus building [114].

### 4.4. Interpretable AI Models

Graph neural networks (GNNs) operating on the pediatric OSA KG enable outcome prediction while preserving structure and context. Heterogeneous GNNs encode node and edge types (patient, observation, questionnaire score, procedure; “has-measurement,” “treated-by,” “precedes”), and temporal GNN variants incorporate event timing and episode boundaries [115]. Tasks include predicting residual OSA after surgery, likelihood of CPAP adherence at three months [116], or risk of neurobehavioral impairment given baseline PSG and comorbidity profile. Training uses stratified, time-split validation to respect temporality; evaluation emphasizes AUROC/AUPRC alongside calibration (Brier score, reliability diagrams) and clinical utility (decision curves) [97,117]. Because inputs are graph-structured, models can naturally integrate multimodal evidence with pediatric-specific semantics, improving transportability relative to flat-feature baselines [115].

Interpretability is delivered through explainable pathways that connect symptoms to interventions and outcomes. Post hoc explainers tailored to graphs (e.g., GNNExplainer, PGExplainer) identify compact subgraphs including specific questionnaire items, obesity status, REM-predominant events, allergy control that were most influential for a prediction [118,119], while counterfactual analysis searches for minimal graph edits that would change the recommendation (for example, “if allergic rhinitis were controlled and BMI-z decreased by 0.5, residual-risk classification would drop below threshold”). These explanations are rendered as human-readable narratives with clickable evidence nodes, aligning with clinical reasoning and supporting shared decision-making with families [120,121]. Crucially, explanations are validated against domain rules encoded in the ontology (age-appropriate thresholds, contraindications), ensuring fidelity to pediatric practice [108]. Uncertainty estimates (e.g., conformal prediction sets) accompany outputs so clinicians can weigh predictions appropriately and prioritize confirmation testing when model confidence is low [122].

Together, these applications illustrate how a pediatric-first KG transforms fragmented data into actionable, auditable intelligence. Clinical teams gain transparent tools for screening, peri-operative planning, and longitudinal management; researchers gain reproducible cohorts, rapid hypothesis testing, and living evidence maps; and model developers gain a semantically rich substrate that supports interpretable, temporally aware learning [123]. The common thread: explicit semantics, provenance, and time ensures that advances in automation translate into trustworthy improvements in pediatric sleep health [124].

### 4.5. Preliminary Feasibility Demonstration

To provide initial empirical support for the proposed knowledge graph (KG) framework, we constructed a small synthetic pilot dataset (*n* = 24) reflecting the multimodal structure of pediatric OSA data described in this architecture, including polysomnography-derived metrics (AHI, oxygen nadir), questionnaire-based symptom burden (PSQ), comorbidities (e.g., obesity, asthma, allergic rhinitis), and longitudinal outcomes following intervention.

To directly address clinically relevant edge cases, we explicitly incorporated discordant phenotypes, including (i) patients with high physiologic severity (AHI ≥ 10) but low reported symptom burden and (ii) patients with low AHI but high symptom burden. These discordant cases are commonly encountered in pediatric sleep medicine and represent a key limitation of existing single-modality models.

**Figure 5:**
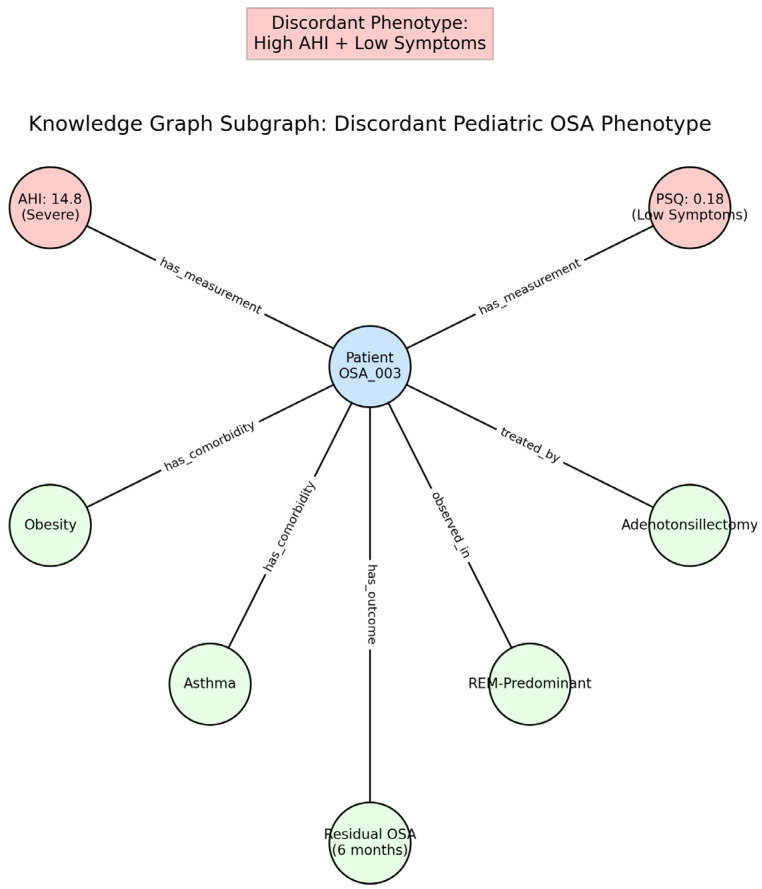
Knowledge graph subgraph illustrating a discordant pediatric OSA phenotype. This example demonstrates a patient with severe physiologic disease (AHI = 14.8 events/h) but low reported symptom burden (PSQ = 0.18). The knowledge graph explicitly represents conflicting clinical signals while preserving contextual relationships, including comorbidities (obesity, asthma), REM-predominant disease, intervention (adenotonsillectomy), and longitudinal outcome (residual OSA at 6 months). Nodes: SNOMED-CT entities; edges: causal relations with probabilistic weights. This structure enables downstream graph-based learning and retrieval methods to identify similar discordant cases and support explainable clinical reasoning.

Within the KG, these cases were represented as structured subgraphs linking measurements, symptom scores, comorbidities, and outcomes. Figure 5 illustrates an example of a discordant phenotype in which severe AHI coexists with low symptom burden. The graph preserves this conflict while contextualizing it with additional features, including obesity, asthma, and REM-predominant events.

To demonstrate graph-based reasoning, we applied a simple similarity-based retrieval over graph-derived feature embeddings. For a representative discordant case, the system identified clinically similar patients sharing features such as REM predominance and obesity, many of whom exhibited persistent disease following intervention. This suggests that the graph structure captures meaningful clinical relationships beyond individual variables.

In addition, we implemented a prototype retrieval-augmented generation (RAG) workflow constrained by the KG. Given a clinician-style query (e.g., “Why does this patient have severe AHI but minimal symptoms?”), the system retrieved relevant subgraph elements and generated an explanation grounded in structured evidence (e.g., REM predominance, obesity, comorbid asthma), demonstrating the feasibility of explainable, graph-constrained reasoning. The RAG architecture requires iterative refinement, as would be expected for any advanced platform.

While this pilot is limited in scale and uses synthetic data, it provides proof-of-concept evidence that the proposed KG + GNN + RAG architecture can (i) represent multimodal pediatric OSA data, (ii) explicitly encode discordant clinical signals, and (iii) support interpretable reasoning over complex patient profiles. These results motivate future validation using real-world, multi-institutional datasets.

## 5. Discussion—Challenges and Future Directions

### 5.1. Data Availability and Quality

The most immediate barrier to a robust pediatric OSA knowledge graph (KG) and its downstream analytics is the scarcity of labeled multimodal datasets that span signals, tabular clinical variables, narratives, and longitudinal outcomes. Pediatric cohorts are small by nature, heterogeneity across developmental stages is high, and clinically adjudicated labels, specifically for nuanced constructs such as REM-predominant obstruction, hypoventilation, or behavioral outcomes, are costly to obtain and often inconsistent across centers [20]. Label noise arises from inter-scorer variability in PSG, evolving scoring manuals, incomplete documentation of comorbidities, and variation in questionnaire administration [125,126]. Domain shift further degrades model transportability: sensors and montage configurations differ across labs; EHR coding practices, care pathways, and referral biases vary across systems; and population mix (e.g., obesity prevalence, craniofacial anomalies) varies by geography and time [127]. Addressing these deficits requires deliberate data governance and engineering: standardized ingestion pipelines with explicit versioning of scoring rules; SHACL- or schema-based validation to enforce semantic and unit consistency [128]; calibrated data-quality metrics (completeness, plausibility, concordance) attached to each node and edge [129]; and targeted labeling campaigns that prioritize high-impact ambiguities identified by disagreement mining. Semi-supervised learning and weak supervision can amplify limited labeled sets, while active learning can focus expert review where model uncertainty is greatest [130]. Finally, benchmark suites (i.e., shared, de-identified task definitions withheld-out external test sets) are essential to evaluate generalization and fairness across ages, racial/ethnic groups, and comorbidity strata [98].

De-identification and data sharing present additional constraints that are particularly acute in pediatrics. Standard date shifting, pseudonymization, and removal of direct identifiers often leave residual re-identification risk when rare phenotypes, detailed timelines, or free-text notes are included [131]. Safe collaboration demands a layered strategy: rigorous de-identification with risk assessment tailored to pediatric rarity [132]; differential privacy for aggregate releases when appropriate [133]; governance frameworks with clear data-use agreements, provenance capture, and audit trails; and technical enablers such as secure data enclaves, federated learning, and federated analytics that allow models to be trained across institutional KGs without raw data leaving local control [134,135]. Synthetic data can help with software testing and education but should not be treated as statistically equivalent to source data without careful utility and privacy evaluation. For free text, structured extraction pipelines must retain bidirectional links to redacted spans so that clinicians can verify assertions without exposing protected information.

### 5.2. Ontology Development

Progress is gated by the scarcity of pediatric OSA-specific semantic standards. General terminology captures many building blocks (e.g., problems, findings, procedures, medications, and measurements) but rarely encodes age-conditioned definitions, developmental considerations, or pediatric scoring nuances central to sleep medicine [136]. A sustainable path forward is a layered ontology that reuses established vocabularies for core entities while adding minimal pediatric extensions expressed in OWL and validated by SHACL shapes [128]. Age-conditioned axioms should be first-class semantics rather than free-text annotations. For example, an AHI of 13 would indicate severe obstructive sleep apnea in a 3-year-old, while this same AHI would represent only mild obstructive sleep apnea in a 19-year-old. Likewise, questionnaire constructs (item-level responses, subscales, cut points) need computable definitions to support reproducible phenotyping and decision support. Each extension must be versioned, provenance-stamped, and accompanied by competency questions that demonstrate clinical utility (e.g., “identify children <10 years with REM-predominant severe OSA and asthma who have residual disease 6–12 months post-adenotonsillectomy”).

Community-driven curation is essential to ensure breadth, depth, and adoption. A multi-stakeholder consortium including sleep clinicians, pediatricians, otolaryngologists, orthodontists, informaticians, and patient advocates should govern change proposals, editorial workflows, and release cadence. Transparent contribution pipelines (issue tracking, pull requests, review boards), reference implementations (public SHACL validators, example SPARQL queries), and alignment artifacts (cross-walks to SNOMED CT, LOINC, RxNorm, HL7 FHIR ValueSets) will lower integration costs [137]. Crucially, the community must commit to longitudinal stewardship: updating axioms when scoring rules evolve, adding new device metrics as technology changes, and retiring deprecated terms with clear migration paths so historical data remain queryable.

### 5.3. LLM Alignment and Trust

Large language models (LLMs) unlock powerful capabilities—entity/relation extraction from notes and reports, clinical summarization, and question answering—but their unconstrained use risks hallucination, subtle clinical inaccuracies, and misplaced confidence. Trustworthy deployment demands alignment strategies that go beyond prompt engineering. Instruction-tuned, pediatric-focused corpora improve baseline adherence to domain style and content, but fine-tuning alone does not guarantee factuality or provenance. A hybrid architecture should bind generation to KG facts through retrieval-augmented generation (RAG), constrained decoding, and post hoc claim checking [102]. In practice, this means pre-restricting the model’s context to a verified, minimal subgraph; enforcing output schemas and ontological constraints during decoding (e.g., preventing age-inappropriate severity labels); and re-parsing generated statements to cross-verify each claim against the KG, flagging unsupported assertions for clinician review. For extraction tasks, span-level provenance and confidence scores must accompany every triple, enabling selective human validation and active-learning loops that prioritize low-confidence or high-impact edges.

Choosing among prompt engineering, fine-tuning, and KG-constrained hybrids is not binary; it is task-specific. Prompt engineering offers speed and flexibility for exploratory tasks and UI iteration but requires strong guardrails and conservative defaults. Supervised fine-tuning on curated pediatric corpora yields better consistency for repetitive tasks (e.g., report summarization) and reduces prompt brittleness. The highest assurance arises from hybrid pipelines that combine RAG with symbolic checks: the KG supplies facts and constraints; the LLM provides language fluency and abstraction; and a verifier reconciles outputs with graph state. Governance should include red-teaming with clinically plausible adversarial prompts, calibration assessment (e.g., expected calibration error, conformal prediction sets), and continuous monitoring for drift, with rollback mechanisms and model cards that disclose training data, intended use, and known failure modes.

### 5.4. Human-in-the-Loop

This adjunctive consultant platform is meant to allow for human-in-the-loop oversight at all stages of the evaluation and decision-making process. We strongly believe that there is no replacement for the physician’s evaluation and the understanding gleaned from the patient–physician relationship and interactions. Essentially, the system would empower the physician at all stages. This information would be used to enhance the platform in ongoing iterations.

### 5.5. Deployment and Adoption

Even the best models fail without thoughtful integration into clinical workflows. The KG and its services should be surfaced through standards-based interfaces (e.g., SMART-on-FHIR apps, CDS Hooks for event-triggered recommendations, and FHIR Subscriptions for longitudinal monitoring) so that decision support appears in the right place, at the right time, with minimal context switching [138]. Human-factors engineering is critical: interfaces must present concise recommendations with evidence paths, uncertainty indicators, and “why this, why now” explanations, while avoiding alert fatigue via configurable thresholds and suppression rules. Implementation should follow iterative Plan-Do-Study-Act cycles with champions in sleep clinics and peri-operative teams, accompanied by training materials and feedback channels that route clinician input back to ontology and model owners. Equity must be monitored explicitly: dashboards should track performance and access across age, sex, race/ethnicity, language, rurality, and payer status, with remediation plans when disparities emerge [98].

Validation and regulatory pathways require the same rigor expected of other pediatric clinical technologies. Before-and-after or stepped-wedge designs can evaluate impact on referral appropriateness, time-to-diagnosis, residual disease detection, and patient-reported outcomes, while external validation across sites quantifies transportability. For software as a medical device, teams should adopt quality and safety frameworks (risk management, software lifecycle, usability engineering, cybersecurity) and maintain algorithm change protocols that pre-specify what can evolve without re-clearance and how performance will be re-verified. Real-world performance monitoring must be continuous: model drift detectors, audit logs for recommendations and overrides, and incident response playbooks for erroneous outputs. Documentation utilizing model cards, datasheets, validation reports, and post market surveillance summaries should be treated as living assets. Adoption hinges on delivering measurable value to clinicians and families: faster, fairer screening; clearer post-operative planning; and more reliable long-term management. By aligning technical advances with governance, safety, and usability, the pediatric OSA KG can translate from promising research into dependable, equitable care.

## 6. Conclusions

Pediatric obstructive sleep apnea (OSA) demands tools that can reconcile heterogeneous evidence, honor developmental nuance, and produce recommendations clinicians can trust. A knowledge graph (KG)-based framework provides that foundation by encoding pediatric OSA as a semantically rigorous, temporally aware, and interoperable representation that integrates signals (PSG), clinical data (EHR), narrative reports, questionnaires, and context such as environment and growth. Anchoring analytics to this graph enables multimodal reasoning, preserves provenance, and supports transparent explanations—transforming AI from narrow, siloed point solutions into a cohesive, explainable system of care. By coupling the KG with graph-aware learning (e.g., GNNs) and KG-constrained generation (RAG + ontological checks), the approach delivers predictions and summaries that are not only accurate, but auditable and aligned with pediatric practice.

Realizing this vision requires a coordinated, open, and sustained collaboration. We call for a multi-stakeholder consortium and standards bodies to co-develop: (i) a pediatric OSA ontology with age-conditioned axioms and SHACL validation; (ii) reference ETL pipelines and federated governance that enable privacy-preserving, multi-site data integration; (iii) benchmark tasks and external test sets spanning diverse populations; and (iv) clinical decision support pilots embedded via SMART-on-FHIR/CDS Hooks with prospective, equity-aware evaluation. Parallel tracks should advance LLM alignment with KG guardrails, publish model cards and change protocols, and establish pathways for regulatory readiness and post-market monitoring. With shared standards, open resources, and rigorous clinical validation, the community can translate the KG framework from concept to practice delivering earlier detection, more precise and equitable treatment selection, and durable improvements in pediatric sleep health.

## Data Availability

No new data was reported in this manuscript.

## Abbreviations

The following abbreviations are used in this manuscript:

AAP	American Academy of Pediatrics
ADHD	Attention-deficit/hyperactivity disorder
AHI	Apnea-hypopnea index
AI	Artificial intelligence
BPAP	Bilevel positive airway pressure
CDS	Clinical decision support
CNN	Convolutional neural network
CPAP	Continuous positive airway pressure
EHR	Electronic health record
ESS-CHAD	Epworth Sleepiness Scale for Children and Adolescents
FHIR	Fast Healthcare Interoperability Resources
GNN	Graph neural network
ICD-10-CM	International Classification of Diseases, Tenth Revision, Clinical Modification
KG	Knowledge graph
LLM	Large language model
LOINC	Logical Observation Identifiers Names and Codes
NLP	Natural language processing
OSA	Obstructive sleep apnea
OWL	Web Ontology Language
PSG	Polysomnography
PSQ	Pediatric Sleep Questionnaire
RAG	Retrieval-augmented generation
REM	Rapid eye movement
SHACL	Shapes Constraint Language
SNOMED CT	Systematized Nomenclature of Medicine—Clinical Terms
SPARQL	SPARQL Protocol and RDF Query Language
UCUM	Unified Code for Units of Measure
